# Advancing hyper-crosslinked materials with high efficiency and reusability for oil spill response

**DOI:** 10.1038/s41598-023-36577-4

**Published:** 2023-06-16

**Authors:** Caleb Karmelich, Zhijian Wan, Wendy Tian, Emma Crooke, Xiubin Qi, Ann Carroll, Kristina Konstas, Colin Wood

**Affiliations:** 1 Energy Business Unit, Commonwealth Scientific Industrial Research Organisation (CSIRO), Kensington, WA 6151 Australia; 2 Manufacturing, Commonwealth Scientific Industrial Research Organisation (CSIRO), Clayton, VIC 3168 Australia; 3 Commonwealth Scientific Industrial Research Organisation (CSIRO), Private Bag 10, Clayton South MDC, VIC 3169 Australia

**Keywords:** Environmental chemistry, Polymer chemistry

## Abstract

Developing materials with high efficiency for recovering oil to mitigate the environmental impact of oil spills has always been a challenging task. A commercial melamine formaldehyde sponge was coated with an optimised superhydrophobic/superoleophilic hyper-crosslinked polymer and applied to the removal of crude oil from oil-in-water emulsions for the improvement of oil spill clean-up processes. The high surface area, porosity, hydrophobicity, and selectivity of oil over water made the hyper-crosslinked polymer coated sponge (HPCS) an ideal sorbent for efficient oil/water separation. The system was able to strip crude oil from water emulsions of 1000 ppm to a negligible level of 2 ppm oil with minimal amounts of the HPCS material. More importantly, the HPCS material could be reused via a simple mechanical compression process, and the uptake capacity was retained over ten cycles. For five cycles of oil adsorption/mechanical compression the HPCS was able to provide water filtrate with oil concentrations of under 15 ppm. This is an effective and economical recovery system, removing the need for consistent solvent washing and drying processes. These results suggest that the HPCS is a promising material for oil/water separation and recovery under challenging conditions.

## Introduction

Oil spills continue to have catastrophic effects on marine ecosystems around the world today^1–3^. Although the technology for response to oil spill incidents is far more effective and efficient than in previous decades, there continues to be a need to develop technologies that improve clean-up processes whilst ensuring that they are less costly to both marine life and the economy^4^. Typically, the oil spill response process consists of skimming to remove oil from the surface of the water, which works well in calm conditions and can remove the bulk of spilled oil^5,6^. Although skimming operations are fast and low cost, they result in the collection and storage of a large amount of oil/water (O/W) mixtures on the response vessel. One of the most difficult tasks in oil spill response is the separation of these O/W mixtures where the oil is dispersed in the water as small droplets or as an emulsion^7^. This can be attributed to the time required to wait for the oil and water to separate, so the sea water must be processed onshore rather than at sea. Returning to port wastes crucial operational time, therefore, there is a need for a reusable material capable of quickly separating the oil-in-water emulsions and increasing the efficiency of oil spill response operations. Ideally the material should be regenerable at sea without requiring solvent-based washing.

The ideal material for oil capture will have optimised surface chemistry, high surface area, porosity, and selectivity towards non-polar molecules^8,9^. Other important features include an easily scalable and inexpensive synthesis as well as facile regeneration of the material at sea^7^. Due to the combined requirements of highly selective surface conditions and low manufacturing cost, practical solutions have so far been difficult to achieve. Microporous materials (materials with micropores < 2 nm in diameter) have been heavily researched for this application and shown to be effective to enhance the separation efficiency during oil spill clean-up processes. Many materials currently being researched include metal meshes^10,11^, membranes^12,13^, aerogels^14,15^, sponges/foams^16–18^, and other engineered porous materials^19,20^. Unfortunately, widespread implementation of these materials is often limited by the scalability and costs of production, as well as poor long-term robustness. In addition, the regeneration processes required, such as extensive solvent washing, contribute negatively to the environment and cost. In order to generate desirable materials for oil spill response, inexpensive substrates such as commercially available polyurethane and melamine sponges are commonly used as a scaffold for functionalised oil adsorption materials because they already contain abundant porosity with high adsorption capacity for both polar and non-polar liquids^7,19,21,22^. Functionalising the sponge by modifying the surface chemistry can allow for selective capture of either polar contaminants with a hydrophilic/oleophobic surface or non-polar contaminants with a hydrophobic/oleophilic surface. The modifications are commonly applied by an in-situ chemical reaction that deposits the reactants on the surface of the sponge, dip coating, vapor deposition or infitration^12,22^. Recently, researchers at the Argonne National Laboratory have developed a technique to grow a thin layer of metal-oxide primer on the interior surface of polyurethane foam, which enabled the functionalised foam to be efficient for capturing oil sheens^22^.

In our previous work we developed a novel method to coat domestic melamine formaldehyde (MF) sponges with hyper-crosslinked polymer (HCP) which provides the sponges with superhydrophobicity for efficient oil separation from water^23^. This work aims to demonstrate the robustness and effectiveness of hyper-crosslinked polymer coated sponge (HPCS) used for the removal of crude oil contaminants in water, especially at concentrations around 1000 ppm. In addition, a simple mechanical compression method is applied to regenerate the HPCS for multiple oil clean-up cycles. Expulsion of captured oils from the sponge is far less costly than recycling methods often applied to sponge materials that involve extensive washing with hazardous solvents followed by a distillation of the solvents to recover oil^24,25^. Therefore, compression is far more viable to offshore applications than other sponge cleaning methods. This work demonstrates improvements to HPCS in terms of scalability and recyclability of the material when stripping oil from water to less than 15 ppm oil content, permitting direct disposal of the filtrate into the ocean.

## Experimental

### Materials

Commercially available melamine formaldehyde sponges manufactured by Mr. Clean were bought from a local store. Dichloro-*p*-xylene (DCX, 98%), iron (III) chloride (anhydrous FeCl_3_, 98%), dichloromethane (DCM, anhydrous > 99.8% contains 40–150 ppm amylene as stabiliser), ethanol (99%) and toluene (99.8%) purchased from Sigma-Aldrich. Dichloromethane (DCM, > 99%) purchased from Rowe scientific. Sylgard-184 (polydimethylsiloxane) (PDMS) and PDMS curing agent were purchased from DOW. Hibernia, Cold Lake, Hebron and Shell ULSF crude oil samples were supplied by Fisheries and Oceans Canada.

### HPCS synthesis and coating

0.5 g of melamine formaldehyde sponge was cut into a cube, cleaned with ethanol and water, and then dried in an oven at 100 °C for 24 h. The white pristine sponge was then immersed in a solution of 0.7 g dichloro-*p*-xylene dissolved in 130 mL of dichloromethane. The sponge was allowed to stir within the solution for 10 min before a separate solution of 0.7 g of FeCl_3_ in 80 mL of dichloromethane was slowly added to initiate a Friedel–Crafts alkylation reaction. The mixture was stirred at room temperature for 20 h at 100 rpm. The hyper-crosslinked polymer-coated sponge (HPCS) was removed from the mixture and washed with water and ethanol before drying overnight in an oven at 80 °C. The dry and clean HPCS was then immersed in a mixture of PDMS and curing agent in a weight ratio of 10:1 in toluene. The weight of PDMS and curing agent used was 3% of the HPCS weight. The PDMS was allowed to cure on the HPCS over 48 h at room temperature. The HPCS was removed from the toluene and dried in an oven at 80 °C for 12 h.

### Characterisation of the synthesised HPCS

Nitrogen physisorption technique was conducted to measure the surface area and pore volume arising from the hyper-crosslinked polymer layer. Prior to the measurement, the HPCS sample was degassed at 110 °C for 24 h under pressure of 1.33 mbar. The measurement was then performed using a Micrometrics 3Flex adsorption analyser under a liquid nitrogen environment. The surface area was calculated based on Brunauer–Emmett–Teller (BET) theory using the adsorption data in the relative pressure range of P/P_0_ = 0.05 to 0.20 at 77.3 K, and the total pore volume was determined based on the amount of nitrogen adsorbed at P/P_0_ = 0.99. Scanning electron microscopy (SEM) was used to image polymer coating on the sponge skeleton using a Zeiss Merlin FESEM with a secondary electron detector.

### Crude oil adsorption experiments and HPCS recycling

The HPCS adsorption capacity was measured by immersing 0.2 g of HPCS in a pure sample of crude oil for 10 min to ensure the sponge was fully saturated with oil. The saturated sponge was removed from the crude oil and quickly weighed to prevent evaporation of the volatile oil components. The experiments were performed in triplicate and the average values were obtained. The adsorption capacity was calculated by the equation: adsorption capacity (*k*) = (*m*_*a*_–*m*_*b*_)/*m*_*b*_, where *m*_*a*_ and *m*_*b*_ represent mass of the sponge after and before the oil uptake experiment. For recycling trials, adsorption and desorption trials were conducted using Hibernia crude oil. The HPCS was immersed in crude oil for 10 min, removed from the oil and weighed, compressed three times, and then placed back into a crude oil sample for the next adsorption trial.

The recycling process of the sponge was accomplished by mechanical compression. After an adsorption trial the sponge was placed within a large 25 mL syringe and compressed to below the 3 mL mark twice. The sponge was then removed from the syringe, weighed, and applied to an adsorption trial.

### O/W emulsion separation experiments

O/W emulsions were prepared by using a DAIHAN Scientific HG-15A homogenizer to prepare a 1000 ppm O/W emulsion. To investigate the efficiency of the HPCS as an oil separator, 500 mL O/W emulsions were prepared with Hibernia crude oil and separated with a 0.3 g sample of HPCS with the dimensions of 2 cm × 2 cm × 4 cm. The O/W separation apparatus was comprised of a reservoir containing the 500 mL 1000 ppm O/W emulsion, with tubing connected to a peristaltic pump which pumped the emulsion at a flow rate of 40 mL/min through a 3D-printed filter holder containing the HPCS filter. The filtrate from the filter sponge was allowed to flow into a separate reservoir for collection.

### Filtrate characterisation

Filtrate samples from O/W separation trials were analysed using total scanning fluorescence (TSF) analysis at CSIRO Kensington and by total remaining hydrocarbon (TRH) analysis at an external analytical company, MPL Laboratories which has NATA accreditation in compliance with ISO/IEC 17025. TSF analysis was carried out using a Varian Cary-Eclipse fluorescence analyser at room temperature. The excitation wavelength range was 220:340 nm, increasing in 2 nm increments and the emission wavelength range was 250:540 nm, increasing in 5 nm increments. The 30 nm lag of the excitation wavelength behind the emission wavelength was to avoid interference due to Rayleigh scattering. TSF analysis was completed by preparing a 1 ppm standard solution of Hibernia crude oil in dichloromethane and using it as the reference point for all filtrate samples. After each adsorption trial a sample of the filtrate water was acquired, and the non-polar components were manually extracted with DCM. The DCM solution was then applied to TSF analysis, the resulting excitation/emission matrix was compared to the 1 ppm standard solution to measure an oil ppm value, the excitation/emission pair used for concentration comparison was Ex 262.03 nm and Em 367.03 nm. Each experiment was run triplicate and the standard deviation of the maximum intensity peak was used in the error calculation. External TRH analysis was carried out by MPL laboratories to verify the TSF results, the concentration values presented in the results are derived from TSF experiments.

## Results and discussion

### HPCS fabrication and characterisation

Previous work with the HPCS material developed an optimised synthesis process which demonstrated the effectiveness of the sponge for separating oil-in-water emulsions with refined mineral oil^23^. The purpose of the HPCS material is for use as a tertiary treatment solution for oil spill clean-up, this research to demonstrates and optimises the process of separating crude oil from water and attaining filtrate with a crude oil concentration less than 15 ppm.

**Figure 1:**
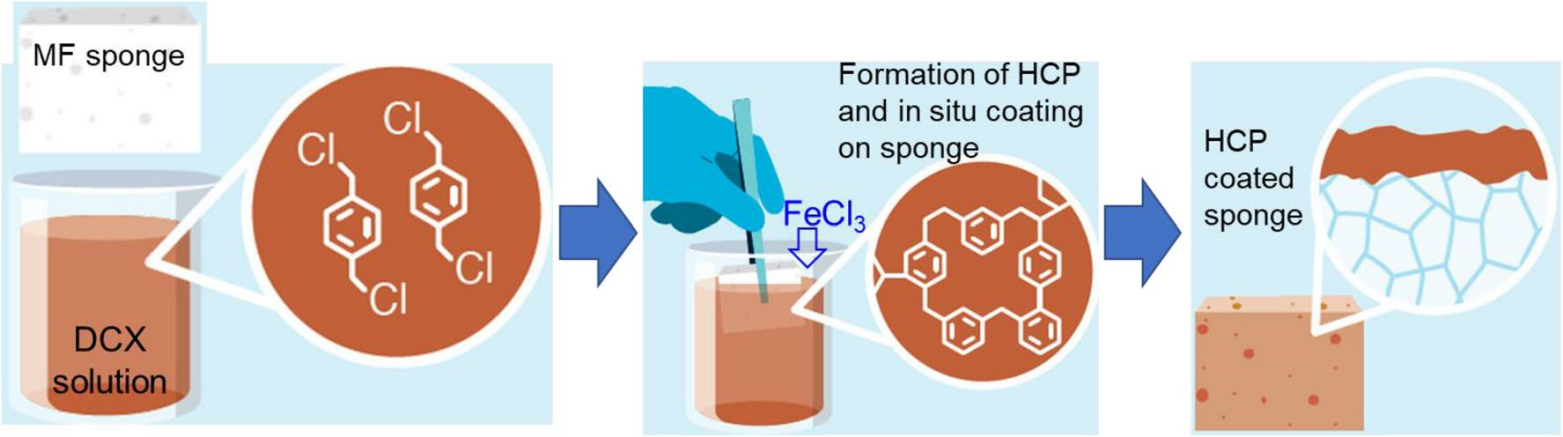
Schematic of synthesising hyper-crosslinked polymer-coated sponge in three simple steps: (i) immersing a pristine MF sponge in a solution of DCX in DCM; (ii) FeCl_3_ is added to the stirring solution to catalyse the hyper-crosslinking reaction of DCX; and (iii) formation a uniform superhydrophobic polymer layer throughout the pores of the MF sponge.

The synthesis process was adopted from our previous work with slight modifications^23^. Shown in Fig. 1 is the schematic for the synthesis of HPCS. A commercially available MF sponge was submerged in DCX solution, followed by the addition of FeCl_3_ to initiate the Friedel–Crafts alkylation reaction to form the hyper crosslinked polymer. Finally, a PDMS curing process was performed to stabilise the HCP on the sponge^26,27^. The optimisation was done with an aim to further decrease synthesis cost, ensuring that the process is scalable and industrially practical. The ratio of the dichloro-*p*-xylene (DCX) and dichloromethane (DCM) solution to MF sponge was decreased to reduce costs. Polydimethylsiloxane (PDMS), a transparent silicone-based protective coating, was applied to the sponge surface and cured as a protective layer to increase robustness of the sponge and ensure the polymer powder remained on the sponge surface. The PDMS also further contributed to the hydrophobicity of the sponge^28^.

**Figure 2:**
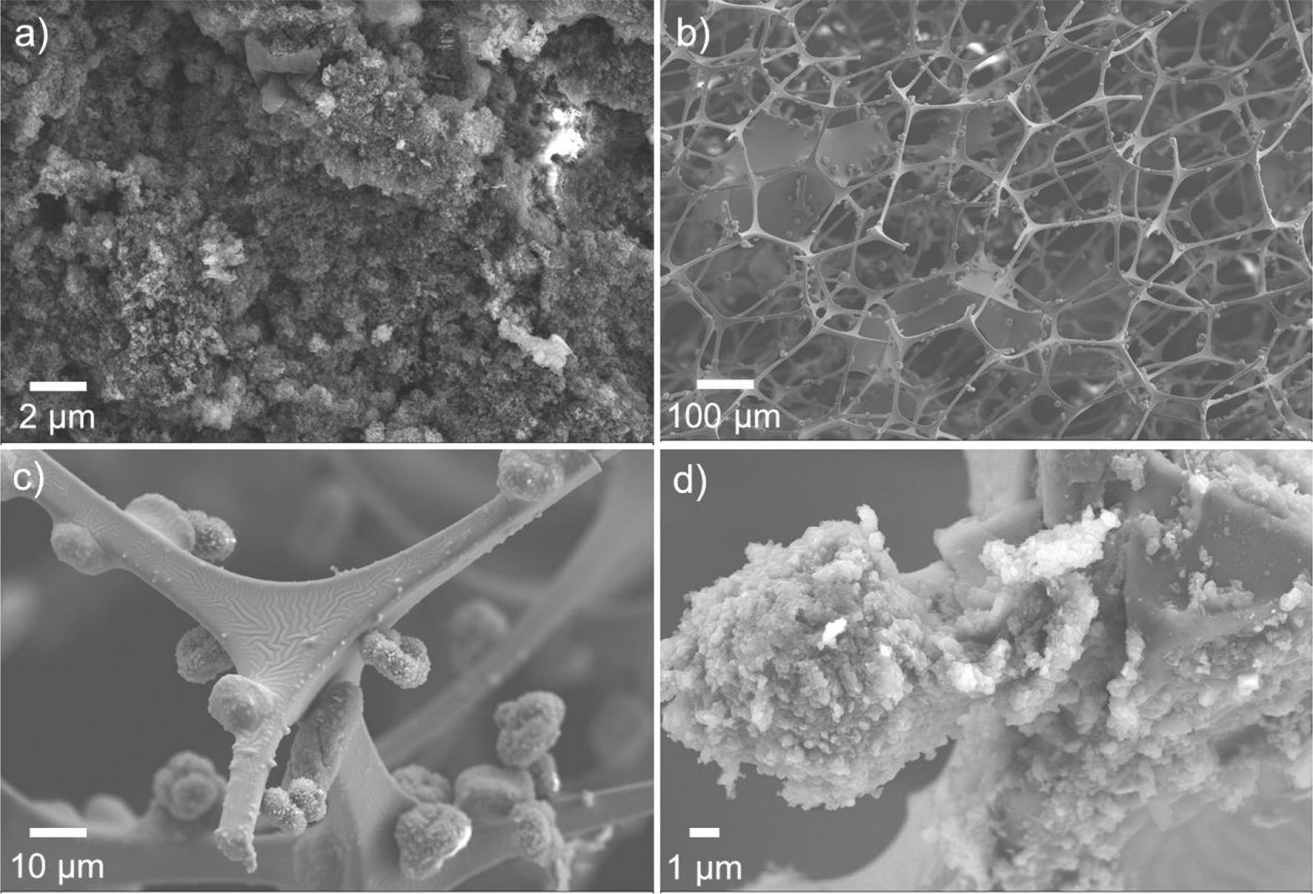
SEM images of (**a**) pristine HCP particles and (**b**–**d**) HCP deposited on the skeleton of sponge.

The resulting sponge was a yellowish-brown colour due to the polymer coating (Fig. S1), slight degradation of the sponge occurred prior to fully coating the DCX on the surface but the MF sponge retained most of its original form. The superhydrophobicity of the HPCS was tested in previous work with contact angles determined to be above 150° with water and 0° with oil^23^. Figure 2 shows the SEM images of the pristine HCP (Fig. 2a) and the MF sponge with HCP coating (Fig. 2b, c). When formed in-situ on the interconnected skeleton of the sponge, the HCP particles were uniformly dispersed on the surface of the skeleton as seen in Fig. 2c, d. This maximises the exposure of HCP particles to oil molecules and thus increases the absorption efficiency. Nitrogen physisorption surface area analysis was performed to determine the surface area of the HPCS material. The sorption isotherms are presented in Fig. S2 and the BET surface area was measured to be 108.75 m^2^/g. It should be noted that the melamine formaldehyde sponge did not contribute to the BET surface area as the macropores of the sponge do not adhere to the BET multilayer gas sorption theory. However, with the hyper-crosslinked polymer coating, the surface area for the sponge was 108.75 m^2^/g, this is an increase from 0 m^2^/g. Although the bulk HCP surface area is often higher than 1000 m^2^/g, the amount of HCP coating on the surface of the MF sponge is limited and so the surface area per gram of material is significantly lower. However, the porosity of the HCP is still present and is significant enough to effect hydrophobicity of the sponge. The average pore diameter of the HPCS coating was determined to be 2.66 nm with pores of diameter between 17 and 300 nm providing a BET surface area of 36.36 m^2^/g. The nanometre-scale pores drastically increase the total surface area of the sponge and should be effective for capture of the small hydrocarbons commonly found in emulsions during oil spill clean-up operations.

### Adsorbed crude oil recovery

**Figure 3:**
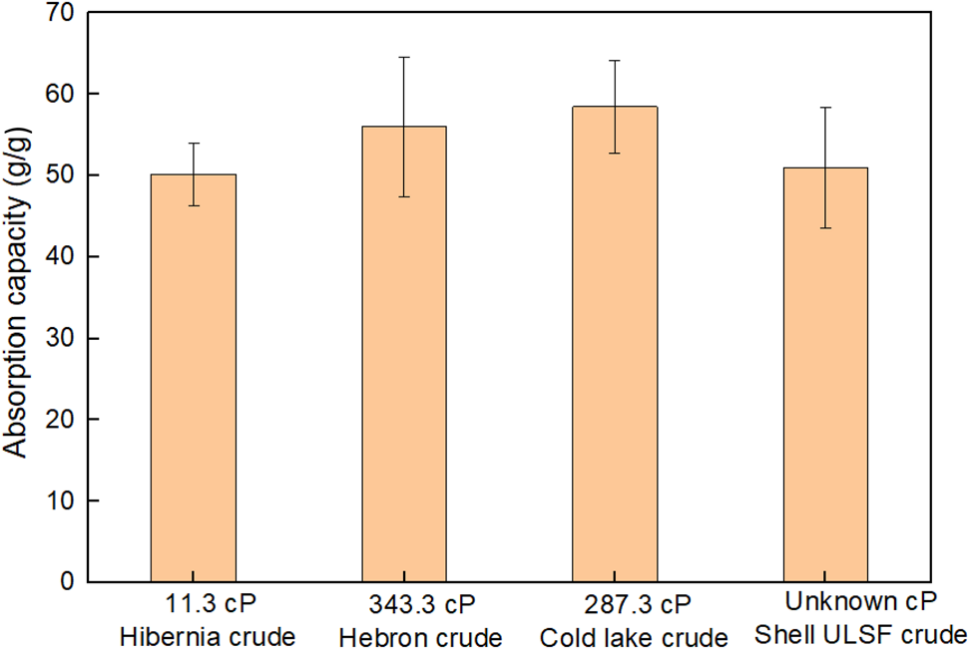
Pure crude oil uptake capacity using HPCS material. Adsorption capacity of HPCS using various crude oil samples from Canada with known viscosity values at 50 °C. All trials were performed in triplicate and the average value is reported.

Four samples of crude oil from Canada (Hibernia, Hebron, Cold Lake and Shell ULSF) were applied to pure crude oil uptake trials. Overall, the uptake capacity of the HPCS was similar between all crude oils, despite the variation in oil viscosity. The oil which resulted in the lowest uptake capacity was the Hibernia crude oil sample at 50.14 ± 3.91 g/g and the highest uptake capacity was with the Cold Lake crude oil with 58.46 ± 5.69 g/g (Fig. 3). The results are similar despite the two oil samples being very different in terms of viscosity, 4.8 cSt for Hibernia crude and 53.0 cSt for Cold Lake crude at 50 °C. The variation in uptake capacity may be due to a difference in hydrocarbon composition of the crude oils rather than viscosity. Cold lake crude oil has a higher volume percentage of aromatics than Hibernia crude oil, however, it has a lower volume percentage of naphthenes and paraffins. This likely contributes to the difference in uptake capacity due to aromatic interactions between the oils and the aromatic groups of the HCP coating^29^. The Shell ultra-low sulphur fuel crude oil was by far the most viscous oil sample used during these trials but the viscosity did not have a significant effect on the uptake capacity of the HPCS as it achieved 50.97 ± 7.38 g/g. The lack of sensitivity of the HPCS to viscosity may be a result of the large variety of pore sizes within the sponge, ranging from large cavities in the MF sponge to the micro and nano-sized pores in the hyper-crosslinked polymer coating. Previous work with HPCS involved measuring uptake with various organic solvents where similar adsorption capacity values were reported, between 40 and 80 g/g for most trialled samples^23^. Other Melamine based sponges are reported to have similar adsorption capacity results ranging from 20 to 130 g/g^30^. Overall, the adsorption capacity of the HPCS is high relative to similar materials, especially considering the low cost and ease of synthesis of the sponge. There are filters used for oil capture that have superior uptake capacity, but they frequently require very expensive and complicated synthetic processes to manufacture. Past research with a variety of pure organic solvents and oils, combined with the data collected here with crude oil mixtures, demonstrates the capability of the HPCS to adsorb a large variety of non-polar compounds with a high loading capacity. Further work to tune the pore sizes of the HPCS could result in better uptake of oils within a particular viscosity range.

### Oil-in-water emulsion separation

Although the crude oil uptake capacity of HPCS was high, a more important characteristic is whether the sponge can quickly and effectively strip water of hydrocarbon contaminants. To test the effectiveness of O/W emulsion separation, experiments using 1000 ppm emulsions of medium to heavy crude oil in water were conducted to mimic conditions that would be encountered during oil spill response operations. The super-hydrophobic polymer coating of the MF sponge repels water and other polar molecules, non-polar hydrocarbons are therefore selectively captured by the HPCS as they are adsorbed within the oleophilic pores. The repelled water is pushed out of the sponge as the emulsion is pumped through the HPCS, ideally this results in a complete separation mixture of oil from water, with an oil concentration in the filtered water less than 15 ppm.

**Figure 4:**
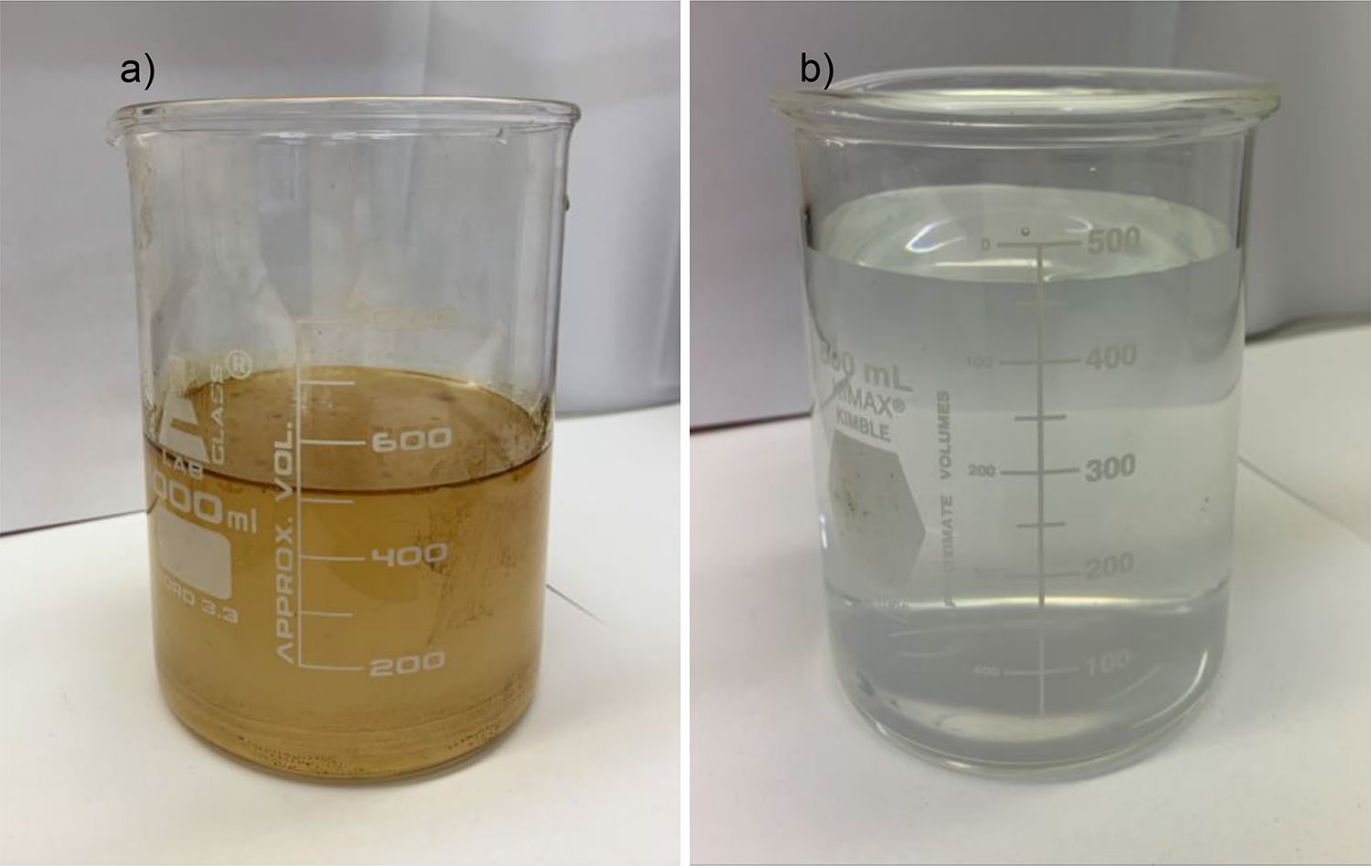
O/W emulsion separation using HPCS. (**a**) 1000 ppm Hibernia crude oil in water before treatment with HPCS. (**b**) Filtrate after 3 cycles of the 1000 ppm O/W emulsion through 0.3 g of HPCS, the filtrate contained 2.35 ppm hydrocarbon content.

Past research with a similar material focussed on using one pure component such as n-hexane or using a sample of refined mineral oil. Crude oil is a mixture of vastly different hydrocarbons, so these trials provide valuable insight about the performance of the HPCS in realistic oil separation applications and provides data on the capture selectivity among different hydrocarbons. Trials were conducted using around 0.25 g of HPCS loaded as a filter inside a cylinder, 500 mL of 1000 ppm O/W emulsion was then pumped through the filter at a flow rate of 40 mL/min. After one cycle through the sponge the filtrate oil concentration was 5.09 ± 1.96 ppm and after 3 cycles through the sponge the filtrate oil concentration was 2.35 ± 2.03 ppm (Fig. 4) as determined by total remaining hydrocarbon (TRH) analysis. Environmental regulations authorise that oily water must not be disposed of directly into the sea if oil concentration is above 15 ppm^31^. The HPCS was able to produce filtrate as low as 2 ppm oil which is well below the 15 ppm oil threshold.

### HPCS recyclability

**Figure 5:**
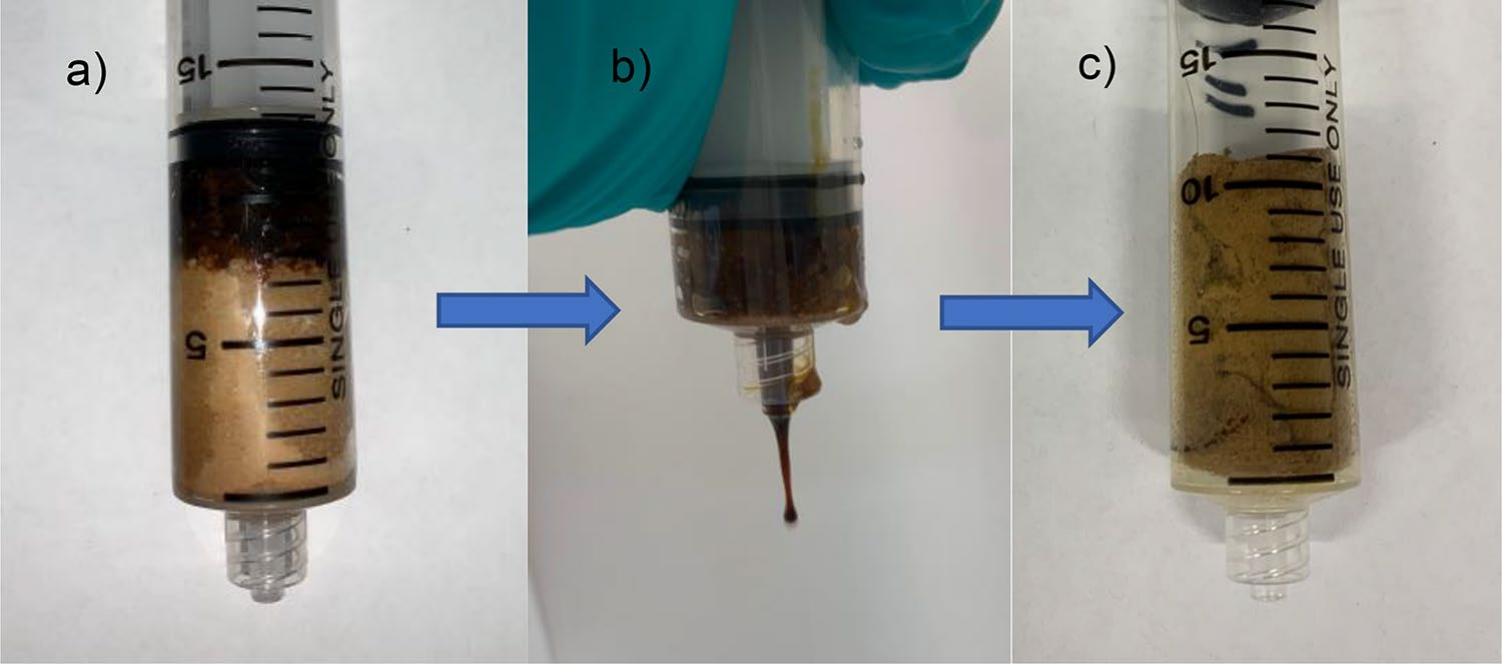
Mechanical compression of HPCS. (**a**) HPCS after adsorption of crude oil. (**b**) HPCS fully compressed. (**c**) HPCS after compression, ready for the next adsorption cycle.

**Figure 6:**
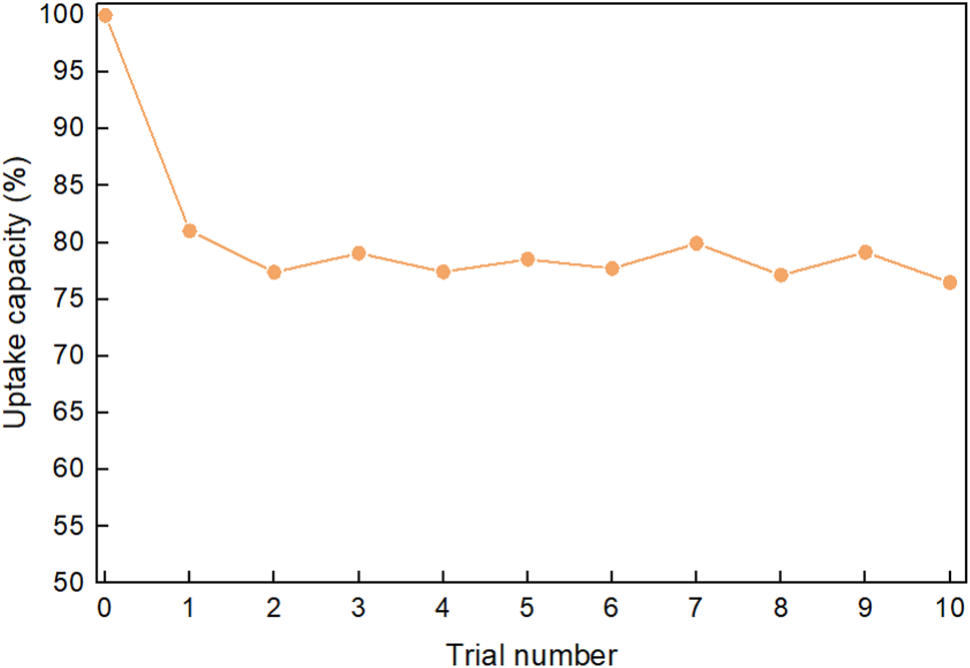
Uptake capacity of HPCS over 10 recycling trials with crude oil. HPCS sample was immersed in Hibernia crude oil and recycled by mechanical compression for 10 cycles. Around 20% capacity was lost after initial saturation, but the uptake capacity was stable over the subsequent 10 adsorption cycles.

As mentioned previously, it is hoped that the process of recycling and collecting the residual oil from the sponge can be made as simple as possible for practical applications. By applying mechanical compression as the method of removing oil and recycling the sponge, the need for washing with solvents and the subsequent distillation of these solvents is removed. This allows for a quicker, cheaper, and more environmentally safe recycling process. With repeated trials using a sample of crude oil, the sponge was recycled by mechanically compressing the sponge (Fig. 5). It was observed that after the original trial (trial 0) with a clean sponge, around 20% of the captured oil remained in the sponge following compression. This result was consistent throughout the trials indicating that without additional cleaning process the sponge will operate at 80% capacity. The oil uptake capacity in trial 1 was 81.04% of trial 0, however this uptake capacity was relatively stable during the subsequent trials, after 10 trails the sponge uptake capacity was still 94.37% of trial 1 (Fig. 6). The HPCS was robust enough to remain stable during the harsh mechanical compression process although the elastic properties of the sponge decreased over time with repeated compressions.

**Figure 7:**
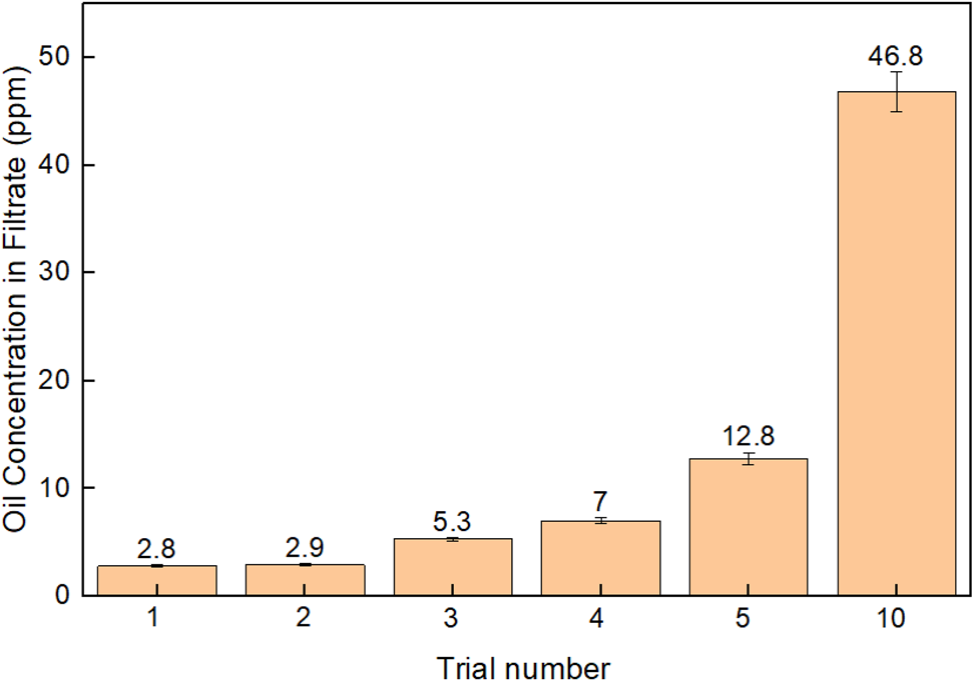
Crude oil concentration of HPCS filtrate following 1000 ppm O/W emulsion separation trials. TSF analysis was used to determine the crude oil concentration in the filtrate from the HPCS sponge over 10 adsorption trials with mechanical compression regeneration. A 1000 ppm oil-in-water emulsion was prepared with Hibernia crude oil for each cycle.

The HPCS demonstrated high ability and stability to capture crude oil, it is also important to analyse the long-term effectiveness of the sponge for separating O/W mixtures and provide water filtrate with an oil concentration of less than 15 ppm. A 0.25 g sponge sample was selected and used as a filter for a 500 mL 1000 ppm O/W emulsion using Hibernia crude oil. After 7 cycles through the sponge, the equivalent of around 28 cm length of filter, the filtrate was collected, and the non-polar components were manually extracted using dichloromethane. The extract was analysed with total scanning fluorescence (TSF), the excitation/emission pair used for concentration comparison was Ex 262.03 nm and Em 367.03 nm, as this was the position of the maximum fluorescence intensity peak for the 1 ppm Hibernia crude oil standard (Table S1). The first five cycles successfully removed enough oil to provide filtrate with less than 15 ppm crude oil, trial 1 had an oil concentration of 2.8 ppm and trial 2 had an oil concentration of 2.9 ppm (Fig. 7). After the fifth separation trial, the oil concentration in the filtrate was 12.8 ppm, all trials were lower than the environmental regulated 15 ppm oil concentration ceiling for sea water disposal. After 5 trials the performance of the sponge appeared to deteriorate rapidly with the filtrate ppm level rising to 46.8 ppm following the tenth trial. Performance may be improved by using a larger and longer sample of sponge, by decreasing the flow rate to increase residence time within the sponge or by increasing the number of cycles through the sponge. Another potential solution for sponge cleanliness is to perform a solvent washing process, typically seen with other sponge materials, after every 5–10 cycles to extend the life of the filter sponge. However, these initial results are encouraging for mechanical regeneration of the materials which has numerous advantages over solvent-based washing. There are some limitations with the TSF method such as the volatility of components in the analyte, recoverability during solvent extraction and instrument drift, so the concentration results were validated by total remaining hydrocarbon (TRH) analysis summarised in Table S2. Equivalent samples to trials 1 and 2 of the TSF experiments were found to have a hydrocarbon concentration of 2.35 ppm and 3.04 ppm, respectively (Table S3). These TRH values were close to the TSF derived samples which were determined to be 2.8 ppm for trial 1 and 2.9 ppm for trial 2. The correlation between TRH and TSF values may deviate further at higher oil concentrations as a quenching effect occurs during TSF analysis, but this was not investigated as the TRH was used to validate the below 3 ppm oil concentration results of the TSF experiments.

Five adsorption trials with ppm levels below the environmental standards is a promising result for the initial investigation with the HPCS sponge. The results demonstrate that although the uptake capacity of the HPCS was stable during the 10 crude oil adsorption trials, the efficiency of the oil separation from water decreased with each cycle. Future research on a larger scale could provide valuable insight about the reliability of the sponge over long periods of time and with a substantial amount of oily water.

**Figure 8:**
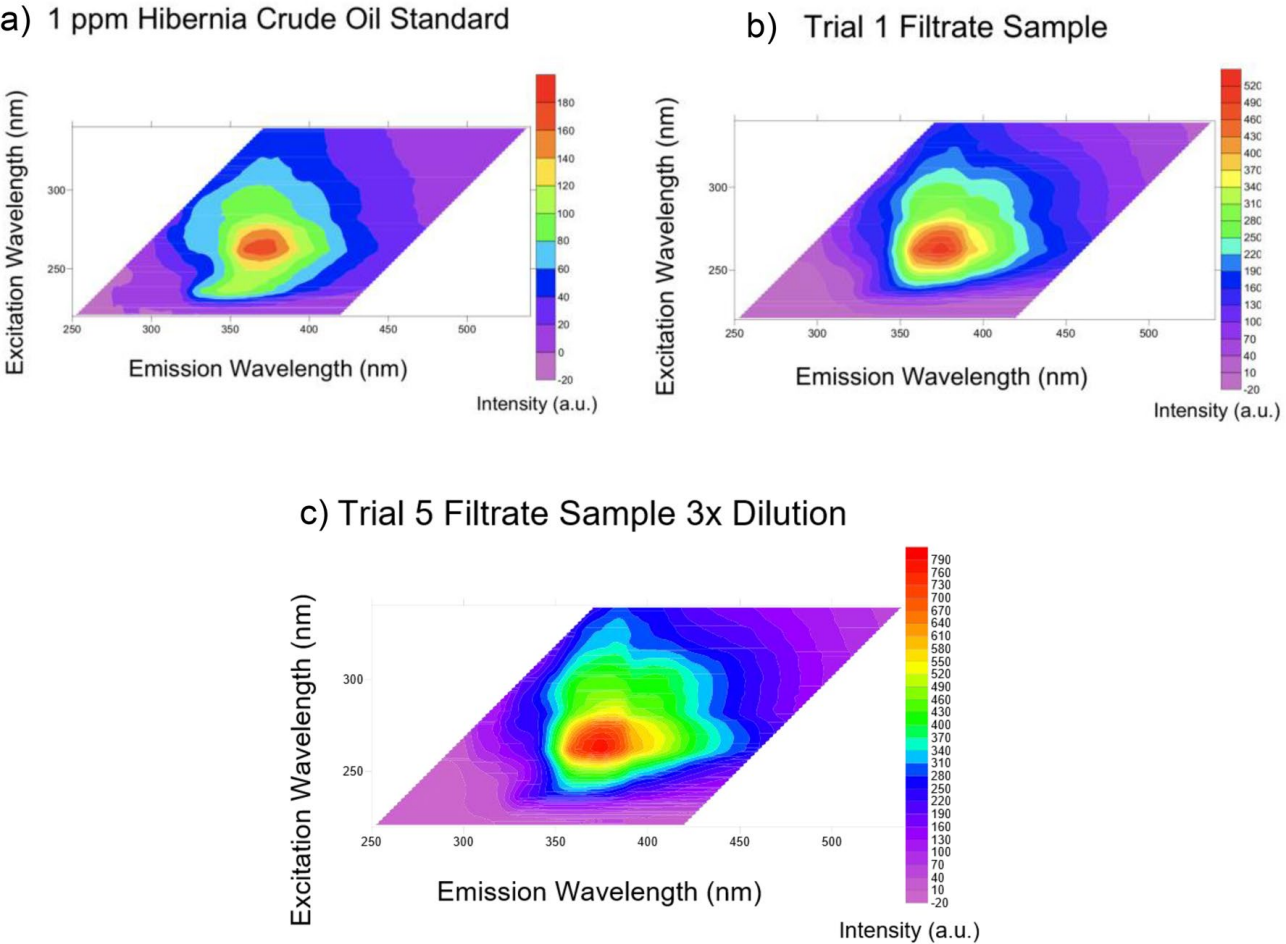
Total scanning fluorescence excitation-emission matrix. (**a**) TSF excitation-emission matrix for 1 ppm standard solution of Hibernia crude oil in DCM. (**b**) TSF excitation-emission matrix for filtrate solution extracted with DCM from the first trial with the HPCS sample. (**c**) TSF excitation-emission matrix for filtrate solution extracted with DCM and a threefold dilution from the fifth trial with the HPCS sample.

Total scanning fluorescence (TSF) analysis was used to quantify the concentration of oil remaining in the filtrate after the separation process and to determine any change in composition of the crude oil following separation to evaluate potential selectivity in oil capture. It was observed that although the intensity of the fluorescence increased after each trial due to rising crude oil concentrations, the distribution of fluorescence on the excitation-emission matrix remained mostly the same (Figs. 8 and S3), indicating the selectivity of the sponge towards different types of oils in the crude oil mixture did not change over time. The position of the peak intensities on the filtrate fluorescence maps did not deviate much from that of the 1 ppm Hibernia crude oil standard, once again indicating the uniform capture of the large variety of oils in the crude oil mixture. Interestingly, although the position of the peak fluorescence on the 1 ppm standard map was the same as the filtrate samples, the contours around the peak of the 1 ppm standard were different. The 1 ppm standard excitation-emission matrix showed a stronger relative intensity towards lower emission wavelengths, 47.48% of the emission wavelength intensity was below or equal to 350 nm. The filtrate samples had low relative intensity below 350 nm and were instead shifted to the right of the map with significant emission fluorescence close to 450 nm. The trial 10 filtrate had 60.07% of emission wavelength intensity above 350 nm (Table S1). This may be indicative of more heavy polyaromatic hydrocarbons being left behind in the filtrate than light polyaromatic hydrocarbons, light hydrocarbons are also more volatile so may be lost during the experimental procedures^32,33^.

## Conclusions

In summary, hyper-crosslinked polymer coated sponges were prepared and optimised for simple ease of scalability and recyclability while maintaining efficient separation of oil from water. The highly versatile sponge was shown to be able to reduce 1000 ppm crude oil-in-water emulsions to as low as 2 ppm with high separation efficiency > 99%. The robustness of the sponge allowed for fast and easy regeneration with mechanical compression without significant loss of uptake capacity over 10 trials. The HPCS was able to strip oil/water mixtures to below environmental disposal standards for at least 5 trials without need of a solvent washing process. The results in this study present a promising prospect for use of HPCS as a large-scale oil separation device during offshore oil spill response operations. Future work may involve applying the HPCS to clean-up operations as a simple solution for the final step of oil spill response, with water volumes upwards of 100 L and fast flow rates. Other potential research areas could involve further improving the ‘green’ synthesis of the HPCS material, as well as developing a tuneable method of synthesis for specific pore sizes within the HPCS.

## Supplementary Information

Supplementary Information.

## Data Availability

The datasets used and/or analysed during the current study available from the corresponding author on reasonable request.
