# Supplementary material for: Advancing hyper-crosslinked materials with high efficiency and reusability for oil spill response
Source: Sci Rep. 2023 Jun 16;13:9779. doi: 10.1038/s41598-023-36577-4 (PMC10276045; doi:10.1038/s41598-023-36577-4)
Supplement: Supplementary file 1 — Supplementary Information. [file 41598_2023_36577_MOESM1_ESM.docx]

**Supporting Information for**

**Advancing Hyper-Crosslinked Materials with High Efficiency and Reusability for Oil Spill Response**

**Caleb Karmelich ^a^, Zhijian Wan ^a^, Wendy Tian ^b^, Emma Crooke ^a^, Xiubin Qi ^a^, Ann Carroll ^a^, Kristina Konstas ^c^ and Colin Wood*^a^**

^a^ Energy Business Unit, Commonwealth Scientific Industrial Research Organisation (CSIRO), Kensington, Western Australia 6151, Australia

^b^ Manufacturing, Commonwealth Scientific Industrial Research Organisation (CSIRO), Clayton, Victoria 3168, Australia

^c^ Commonwealth Scientific Industrial Research Organisation (CSIRO)*,* Private Bag 10, Clayton South MDC, VIC 3169, Australia

*Corresponding Author:

Dr. Colin Wood

Email: [colin.wood@csiro.au](mailto:colin.wood@csiro.au)

Tel: +61-8-64368701


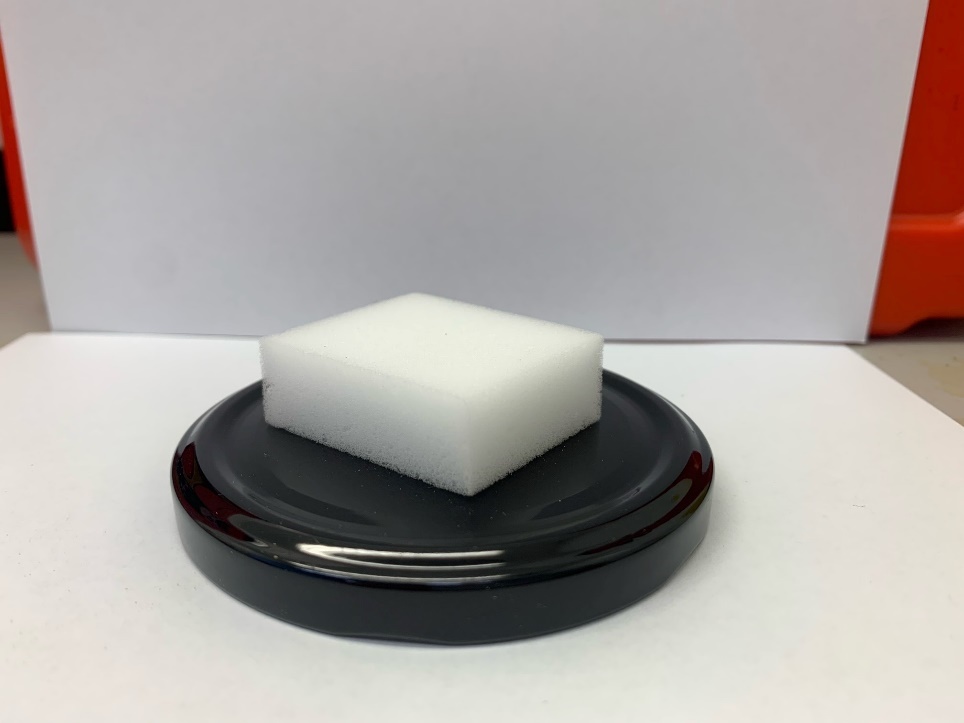

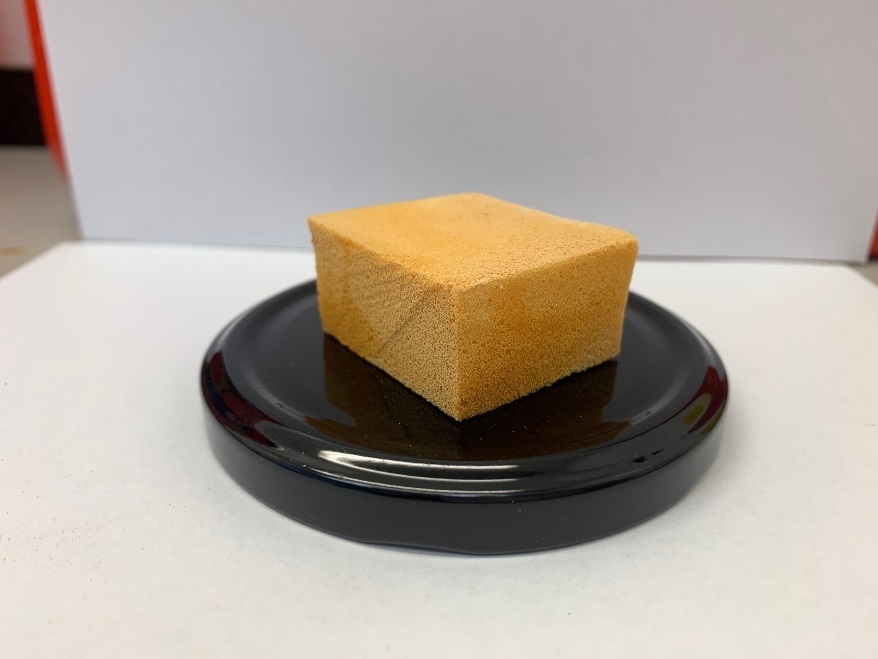


**a)**

**b)**

**Figure S1. a,** pristine melamine formaldehyde sponge. **b,** sponge after super-hydrophobic polymer coating on the surface (HPCS).


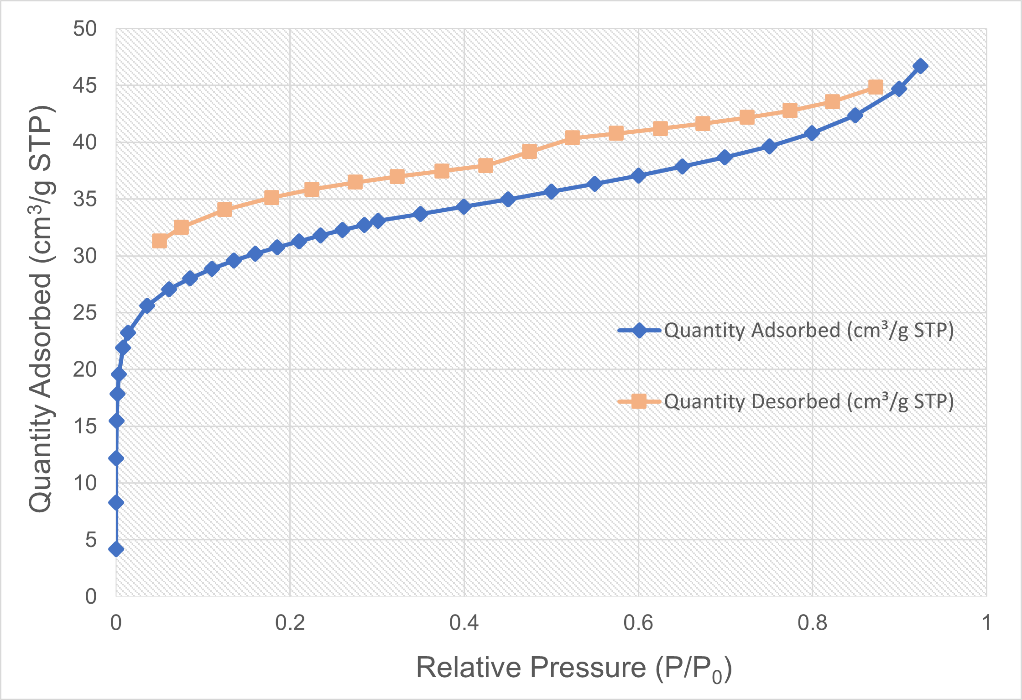

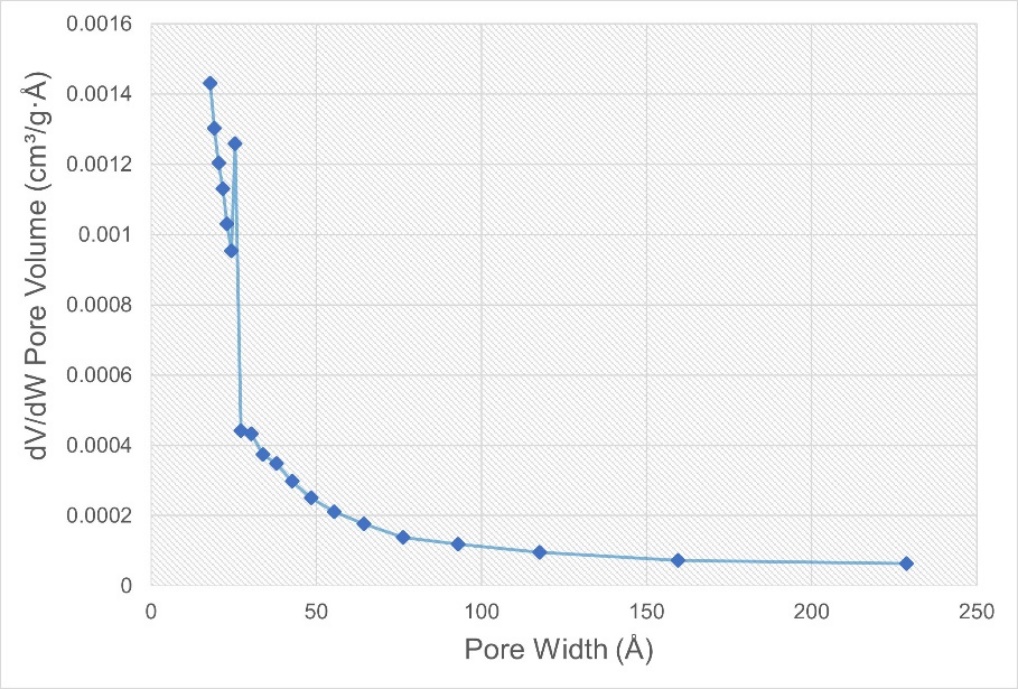


**b)**

**a)**

**Figure S2.** **a,** N_2_ adsorption and desorption isotherms for HPCS material at 77.3 K. **b,** Pore size distribution of HPCS from N_2_ adsorption isotherm.


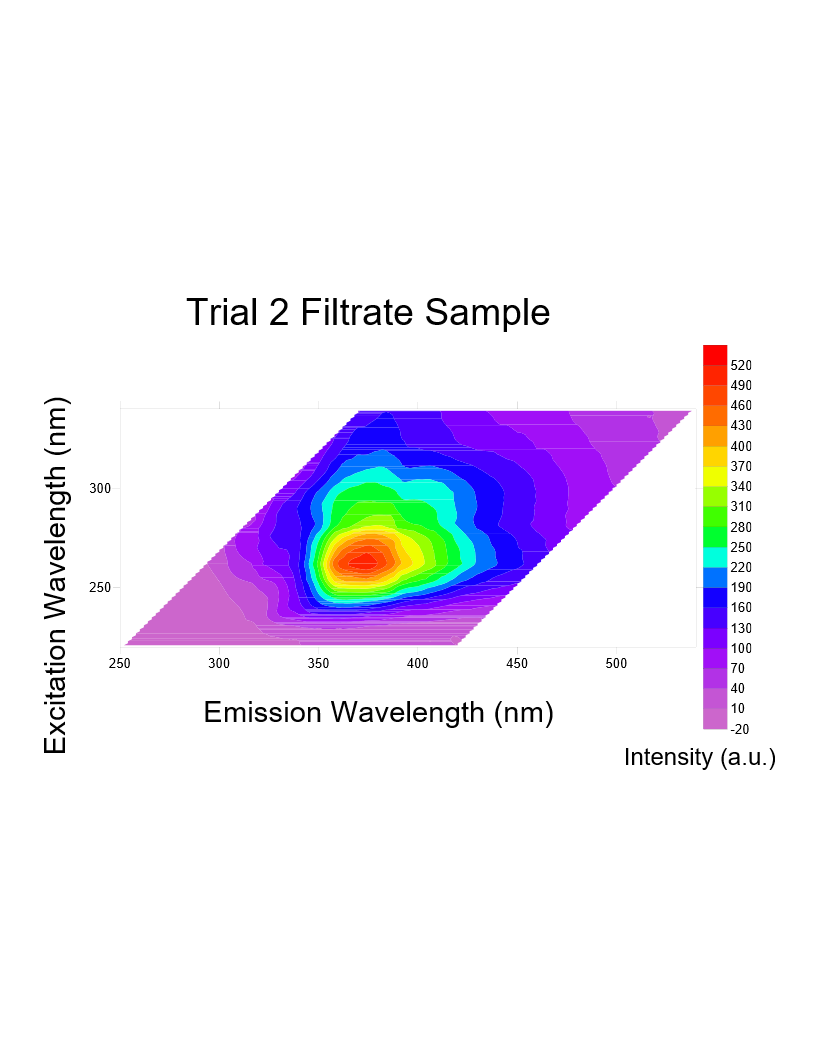

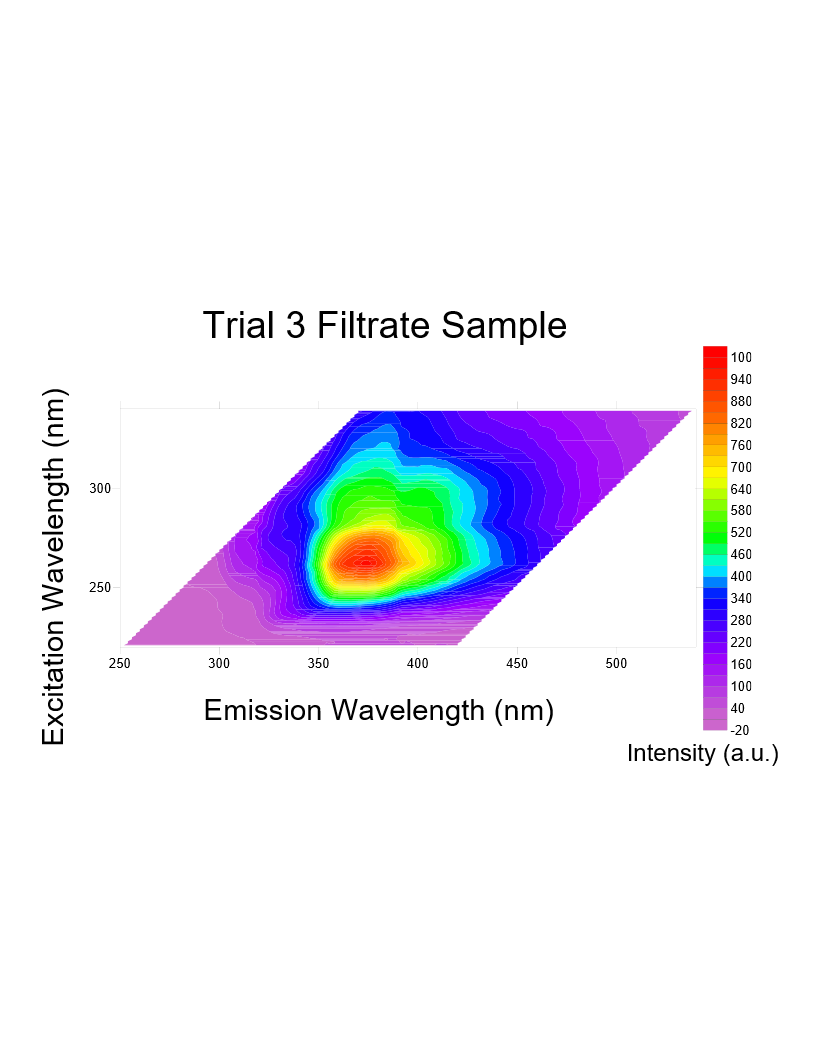


**a)**

**b)**


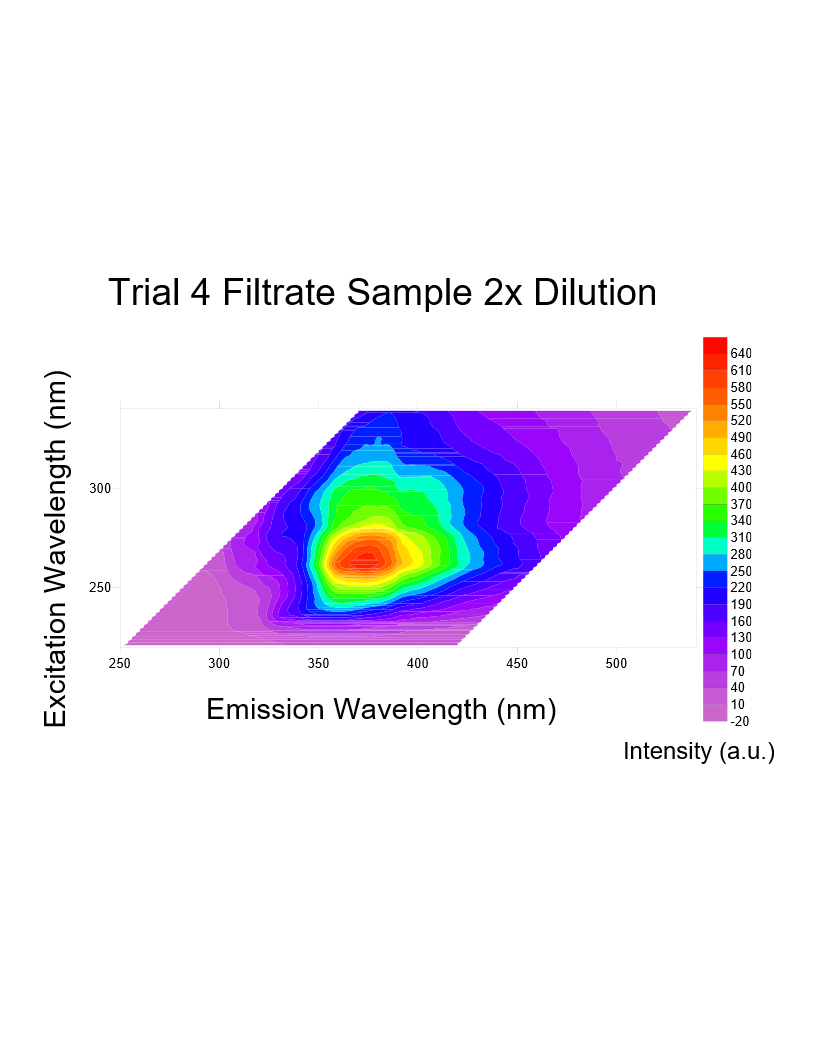

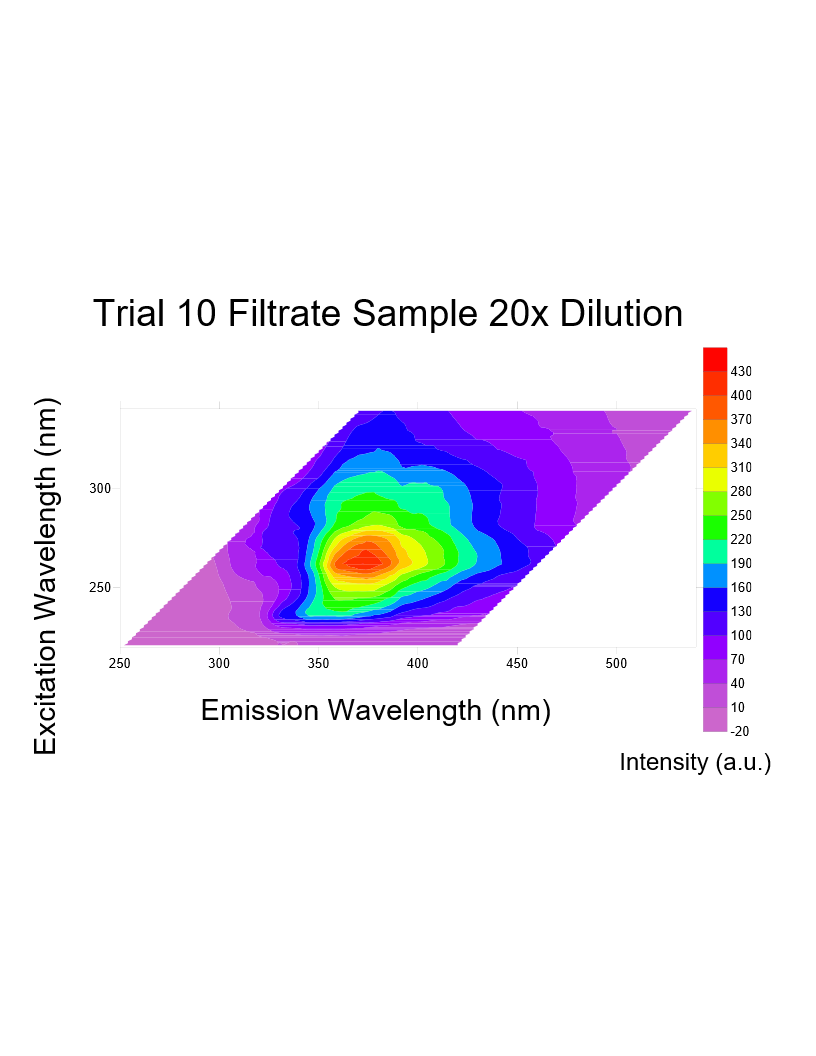


**d)**

**c)**

**Figure S3.** Total scanning fluorescence excitation-emission matrix plots of filtrate samples from separation trials 2 **(a)**, 3 **(b)**, 4 **(c)** and 10 **(d)**.

**Table S1.** Total scanning fluorescence intensity comparison data. Excitation λ 262.03 nm and Emission λ 367.03 nm point chosen as comparison marker because that was a position of maximum emission intensity for the 1 ppm standard solution of the Hibernia crude oil sample.

| Adsorption Trial | Extract Fluorescence Intensity (a.u.) at Excitation λ 262.03 nm and Emission λ 367.03 nm | Total emission intensity below or equal to λ 350 nm | Total emission intensity above λ 350 nm |
| --- | --- | --- | --- |
| 1 ppm Hibernia crude oil standard in DCM | 178.45 | 47.48% | 59.52% |
| 1 | 492.82 | 40.88% | 59.12% |
| 2 | 512.33 | 40.42% | 59.58% |
| 3 | 947.48 | 40.52% | 59.48% |
| 4 | 1252.25 | 41.92% | 58.08% |
| 5 | 2285.89 | 40.20% | 59.80% |
| 10 | 8342.00 | 39.93% | 60.07% |

**Table S2.** Composition of oil in filtrate samples from total remaining hydrocarbon (TRH) analysis.

| **Components** | **Trial 1 Filtrate** | **Trial 2 Filtrate** |
| --- | --- | --- |
| TRH C_6_ - C_9_ µg/L | 770 | 460 |
| TRH C_10_ - C_14_ µg/L | 820 | 1000 |
| TRH C_15_ - C_28_ µg/L | 600 | 1200 |
| TRH C_29_ - C_36_ µg/L | 160 | 380 |
| Benzene µg/L | 19 | 4 |
| Toluene µg/L | 140 | 51 |
| Ethylbenzene µg/L | 47 | 35 |
| *m*+*p*-xylene µg/L | 140 | 110 |
| *o*-xylene µg/L | 88 | 69 |
| Naphthalene µg/L | 52 | 53 |
| Acenaphthylene µg/L | <0.3 | <0.3 |
| Acenaphthene µg/L | <0.6 | <0.8 |
| Fluorene µg/L | 2.1 | 2.7 |
| Phenanthrene µg/L | 1.4 | 2.8 |
| Anthracene µg/L | <0.1 | <0.1 |
| Fluoranthene µg/L | <0.1 | <0.1 |
| Pyrene µg/L | <0.1 | <0.1 |

**Table S3.** Total remaining hydrocarbon (TRH) analysis results for fresh HPCS sample (Trial 1) and after one regeneration cycle (Trial 2).

|  | TRH C_6_ – C_9_ ± 0.01 ppm | TRH C_10_ – C_14_ ± 0.05 ppm | TRH C_15_ – C_28_ ± 0.10 ppm | TRH C_29_ – C_36_ ± 0.10 ppm |
| --- | --- | --- | --- | --- |
| Trial 1 | 0.77 ppm | 0.82 ppm | 0.60 ppm | 0.16 ppm |
| Trial 2 | 0.46 ppm | 1.00 ppm | 1.20 ppm | - 1. ppm |
